# Amniotic fluid embolism rescued by venoarterial extracorporeal membrane oxygenation

**DOI:** 10.1186/s13054-022-03969-3

**Published:** 2022-04-07

**Authors:** Sarah Aissi James, Thomas Klein, Guillaume Lebreton, Jacky Nizard, Juliette Chommeloux, Nicolas Bréchot, Marc Pineton de Chambrun, Guillaume Hékimian, Charles-Edouard Luyt, Bruno Levy, Antoine Kimmoun, Alain Combes, Matthieu Schmidt

**Affiliations:** 1 Service de Médecine Intensive-Réanimation, Institut de Cardiologie, APHP Sorbonne Université Hôpital Pitié–Salpêtrière, 75013 Paris, France; 2 Université de Lorraine, CHRU de Nancy, Institut Lorrain du Cœur Et Des Vaisseaux, Service de Médecine Intensive-Réanimation, U1116, FCRIN-INICRCT, Nancy, France; 3 Institute of Cardiometabolism and Nutrition, Sorbonne Université, INSERM, UMRS_1166-ICAN, 75013 Paris, France; 4 Service de Chirurgie Cardiaque, Institut de Cardiologie, APHP Sorbonne Université Hôpital Pitié–Salpêtrière, 75013 Paris, France; 5 Department of Gynaecology and Obstetrics, Groupe Hospitalier Pitié-Salpêtrière, CNRS UMR 7222, INSERM U1150, Sorbonne Universités, Paris, France; 6 Sorbonne Université, GRC 30, RESPIRE, Assistance Publique-Hôpitaux de Paris (APHP) Hôpital Pitié-Salpêtrière, Paris, France; 7 Service de Medecine Intensive Reanimation, iCAN, Institute of Cardiometabolism and Nutrition, Hôpital de la Pitié–Salpêtrière, 47, bd de l’Hôpital, 75651 Paris Cedex 13, France

**Keywords:** Extracorporeal membrane oxygenation, Amniotic fluid embolism, Cardiogenic shock, Disseminated intravascular coagulopathy, Outcomes

## Abstract

**Background:**

Amniotic fluid embolism (AFE) is a rare but often catastrophic complication of pregnancy that leads to cardiopulmonary dysfunction and severe disseminated intravascular coagulopathy (DIC). Although few case reports have reported successful use of venoarterial extracorporeal membrane oxygenation (VA-ECMO) with AFE, concerns can be raised about the increased bleeding risks with that device.

**Methods:**

This study included patients with AFE rescued by VA-ECMO hospitalized in two high ECMO volume centers between August 2008 and February 2021. Clinical characteristics, critical care management, in-intensive care unit (ICU) complications, and hospital outcomes were collected. ICU survivors were assessed for health-related quality of life (HRQL) in May 2021.

**Results:**

During that 13-year study period, VA-ECMO was initiated in 54 parturient women in two high ECMO volume centers. Among that population, 10 patients with AFE [median (range) age 33 (24–40), SAPS II at 69 (56–81)] who fulfilled our diagnosis criteria were treated with VA-ECMO. Pregnancy evolved for 36 (30–41) weeks. Seven patients had a cardiac arrest before ECMO and two were cannulated under cardiopulmonary resuscitation. Pre-ECMO hemodynamic was severely impaired with an inotrope score at 370 (55–1530) μg/kg/min, a severe left ventricular ejection fraction measured at 14 (0–40)%, and lactate at 12 (2–30) mmol/L. 70% of these patients were alive at hospital discharge despite an extreme pre-ECMO severity and massive blood product transfusion. However, HRQL was lower than age-matched controls and still profoundly impaired in the role-physical, bodily pain, and general health components after a median of 44 months follow-up.

**Conclusion:**

In this rare per-delivery complication, our results support the use of VA-ECMO despite intense DIC and ongoing bleeding. Future studies should focus on customized, patient-centered, rehabilitation programs that could lead to improved HRQL in this population.

Amniotic fluid embolism (AFE) is a rare but often catastrophic complication of pregnancy, occurring in 1.9–2.5 per 100,000 maternities. With a maternal mortality rate ranging from 11 to 43% [1], AFE has become one of the main direct causes of maternal death in developed countries. The pathophysiology of AFE involves disruption of the maternal-placental interface, allowing entry of fetal and amniotic components into the maternal circulation, which induces a massive release of mediators leading to a proinflammatory and procoagulant reaction [2]. Diagnosis is often performed after the exclusion of other peripartum complications. Sudden onset of cardiovascular collapse, cardiac arrest, acute respiratory failure, and fulminant disseminated intravascular coagulopathy (DIC) during delivery are the main clinical signs of the most severe forms. Because the cardiopulmonary dysfunction associated with AFE is typically self-limited, venoarterial extracorporeal membrane oxygenation (VA-ECMO) support has been reported in severe forms [3, 4]. However, the rarity of the condition, and the difficulties to diagnose AFE, make it particularly challenging to study. To date, data available on this specific population rescued by VA-ECMO were mainly described in single-case reports [3, 4] and concerns can be raised about the increased bleeding risks with ECMO in that context.

The objectives of this multicenter, retrospective study were (1) to report outcomes of ECMO-treated AFE; (2) to describe their critical care management and in-ICU complications; and (3) to report long-term maternal health-related quality of life (HRQOL).

## Methods

### Study design and patients

This study included patients with AFE rescued by VA-ECMO hospitalized in two university tertiary medical centers between August 2008 and February 2021. The medical ICUs from Pitie Salpetrière hospital, Paris, and the University hospital of Nancy, France are among the largest ECMO centers in France with more than 100 ECMO cases per year. All consecutive pregnant women who received ECMO during the delivery period were screened. Patients who fulfilled the criteria proposed by Clark et al. [5] for AFE diagnosis were included. Briefly, these criteria combine (1) sudden onset of cardiorespiratory arrest or both hypotension and respiratory compromise; (2) documentation of overt DIC; (3) clinical onset during labor or within 30 min of delivery of the placenta and (4) no fever during labor [5]. DIC was diagnosed using the Modified International Society on Thrombosis and Hemostasis scoring system for overt disseminated intravascular coagulation in pregnancy, which uses platelet count, prothrombin time, and fibrinogen level. A score ≥ 3 being compatible with overt DIC in pregnancy [6]. According to French research methodology MR004, this work was considered a non-interventional retrospective study and therefore only needed information from the survivors. The National Commission for Informatics and Liberties (CNIL) approved this study (no.1950673). Survivors gave oral consent to participate in the telephone interview conducted by the same investigator (S.AJ).

### Data collection

Clinical characteristics of the parturient included gestational age, gravidity and parity status, body mass index, previous medical history, reported AFE risk factors, type of delivery, and anesthetic procedure. Besides, we described the clinical presentation of AFE, the value of serum insulin-like growth factor binding protein-1 (IGFBP-1) (if quantitative dosage available), the presence of fetal material and squamous cells in the maternal bronchoalveolar lavage, and the management of peri-delivery hemorrhage. The following variables were recorded during the first 24 h of ECMO implantation: Simplified Acute Physiology Score (SAPS) 2 [7] and Sepsis-Related Organ Failure Assessment (SOFA) score [8]; pre-ECMO inotrope score defined as the dose of dobutamine (μg/kg/min) + (dose of epinephrine [μg/kg/min] + dose of norepinephrine [μg/kg/min]) × 100 [9]; implantation of ECMO under cardiopulmonary resuscitation; use of intra-aortic balloon pump; blood-gas analyses; and blood lactate.

### Outcome variables

Main outcome variables included survival to ICU discharge, and long-term survival (evaluated in May 2021). Other outcome variables included days under ECMO therapy; time on mechanical ventilation after ECMO implantation; and ICU length of stay. ECMO-associated complications were monitored: leg ischemia, femoral hemorrhage due to arterial laceration, deep-vein or inferior vena cava thrombosis, cannula insertion-site infection, ischemic or hemorrhagic stroke, cardiac tamponade, or other technical problems. Lastly, the number of packs of red blood cells, fresh frozen plasma, and platelets transfused were noted.

### Long-term outcome variables

Patients alive in May 2021 were evaluated for their Health-related quality of life psychologic status during a telephone interview. To assess HRQOL, patients were asked to complete the French version of the Short-Form 36 (SF-36; based on the Medical Outcome Study Survey) questionnaire during a phone call explaining the purpose and objectives of the study. This standardized, widely used questionnaire has been validated for the French population [10]. Its 36 items are combined to evaluate eight domains (physical functioning, role-physical, bodily pain, general health, vitality, social functioning, role-emotional, and mental health). The aggregate physical and mental component summary measures were then computed as recommended by the developers [10, 11]. Our patients’ mean SF-36 levels were compared with those obtained for French age- and sex-matched controls with no adverse health conditions [10], those who had a venovenous (VV) ECMO for a severe acute respiratory distress syndrome (ARDS) [12], and survivors after a septic shock rescued by VA-ECMO [13].

Post-traumatic stress disorder (PTSD)-related symptoms were assessed with the Impact of Event Scale consisting of 15 questions divided into two subscales: intrusion (seven items) and avoidance (eight items) [14]. An impact of event scale score ≥ 30 defined a patient at risk for PTSD [14]. Lastly, other long-term outcome variables included the resumption of professional activity, new pregnancy, and child health status.

### Patient management during ECMO

The detailed management of patients under VA-ECMO was described previously [15, 16]. Briefly, pump speed was adjusted to obtain a blood flow of 3–5 L/min. Intravenous unfractionated heparin was not used in case of DIC, platelets count < 50,000 G/l, or in case of bleeding. For highly unstable patients diagnosed with refractory cardiogenic shock at other hospitals, our institution’s Mobile Circulatory Assistance Units traveled rapidly to primary care hospitals with a portable ECMO system, installed the device before refractory multiple organ failure or cardiac arrest took hold, and then transported the patient to our tertiary cares. The early intra-aortic balloon pump-ECMO combination is currently used to protect against hydrostatic pulmonary edema in peripheral VA-ECMO-assisted patients in case of lack of arterial pulsatility or aortic Velocity Time Integral < 5 cm.

### Statistical analyses

This study followed CONSORT recommendations for reporting cohort studies (STROBE statement) [17]. Data are presented as n (%) or median (range).

For comparisons of patients’ mean SF-36 scores with those of their age- and sex-matched control subjects, or with other populations rescued by ECMO, paired t-tests or Wilcoxon tests were used. *p* ≤ 0.05 defined statistical significance. Analyses were computed with StatView v5.0 (SAS Institute Inc., Cary, NC, USA) software.

## Results

### Study population

During that 13-year study period, VA-ECMO was initiated in 2857 patients, whose 54 were during the peripartum period. Among that population, 10 patients with AFE, who fulfilled our diagnosis criteria [5] were treated with VA-ECMO. None of them had cardiovascular or respiratory history. Their main characteristics are reported in Table 1. Briefly there median (range) age was 33 (24–40) with a pregnancy evolving for 36 (30–41) weeks and a SAPS II at 69 (56–81) at ICU admission. Reported AFE risks factors were frequent with maternal age > 35 years, caesarian section delivery, instrumental assisted delivery, and gestational diabetes being reported in 60, 40, 30, and 30% of our cohort, respectively.

**Table 1:** Clinical characteristics of patients with confirmed amniotic fluid embolism rescued by VA-ECMO

	*N* = 10
Age, years	33 (24–40)
Body mass index, kg/m^2^	27 (21–35)
SAPS 2 score	69 (56–81)
SOFA score	13 (9–17)
Parity, n	2 (1–4)
Gestation, weeks	36 (30–41)
*Amniotic embolism risks factors*	
Maternal age > 35 years	6 (60)
Pre-eclampsia	1 (10)
Placenta insertion abnormalities or hydramnios	2 (20)
Multiple pregnancy	0 (0)
Medically induced labor	3 (30)
C-section delivery	4 (40)
Instrumental assisted delivery	3 (30)
Gestational diabetes	3 (30)
*Obstetric and anesthetic procedure*	
Cesarean section	4 (40)
Vaginal delivery	6 (60)
Emergency procedure	4 (40)
Epidural analgesia	9 (90)
General anesthesia	1 (10)
*Clinical presentation*	
Cardiac arrest	7 (70)
Cardiovascular collapse	9 (90)
Acute respiratory failure	3 (30)
Disseminated intravascular coagulation	10 (100)
Modified ISTH score *	3.5 (3–5)
Altered mental status or seizures	1 (10)
Fetal distress	4 (40)
*Amniotic fluid embolism markers*	
Serum insulin-like growth factor binding protein-1 > 150 μg/L ^§^	5 (62)
Fetal material or squamous cells in the maternal BAL ^£^	5 (62)

Data are expressed as n (%) or median (range)

*Modified International Society on Thrombosis and Hemostasis scoring system for overt disseminated intravascular coagulation in pregnancy use platelet count, prothrombin time, and fibrinogen level. A score ≥ 3 being compatible with overt DIC in pregnancy [5]

^§^Available in 8 patients

^**£**^Available in 8 patients

*BAL* bronchoalveolar lavage, *ICU* intensive care unit, *ISTH* International Society on Thrombosis and Hemostasis, *SOFA* Sequential Organ Failure Assessment, *SAPS II* Simplified Acute Physiology Score, *VA-ECMO* venoarterial extracorporeal membrane oxygenation

Refractory cardiovascular collapse and cardiac arrest, occurring during labor or just after delivery were the most frequent clinical presentation. Besides, all patients had DIC and peripartum hemorrhage. Ongoing severe coagulopathy and post-partum hemorrhage lead to hemostatic procedures and surgical hysterectomy in 70 and 50% of patients. Noticeably, one patient had altered mental status whereas fetal distress was reported in 4 cases. Serum IGFBP-1, performed at different times after the first symptoms, was > 150 μg/L in 5/8 patients whereas fetal material or squamous cells were found in 5 out of 8 bronchoalveolar samples performed in these patients.

All patients received a VA-ECMO initiated by the Mobile Circulatory Assistance Units with a switch to a veno-arteriovenous ECMO in one patient. Seven patients had a cardiac arrest before ECMO, whom two were cannulated under cardiopulmonary resuscitation. Pre-ECMO hemodynamic was severely impaired with an inotrope score at 370 (55–1530) μg/kg/min, a severe left ventricular ejection fraction measured at 14 (0–40)%, and lactate at 12 (2–30) mmol/L.

### Short- and long-term outcomes

**Fig. 1:**
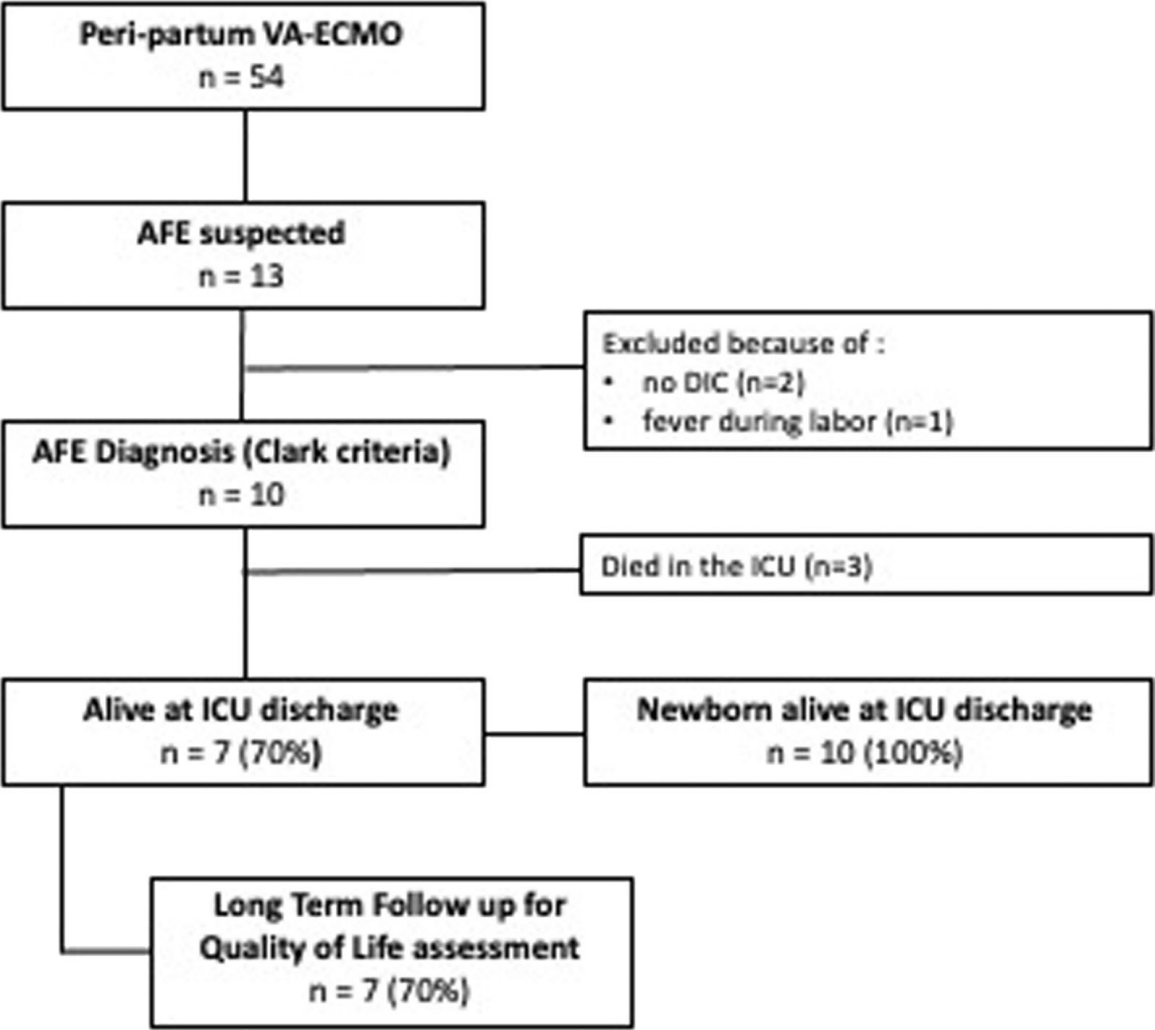
Study flowchart. *AFE* amniotic fluid embolism, *VA-ECMO* venoarterial extracorporeal membrane oxygenation, *ICU* intensive care unit

Complications during ECMO are listed in Table 2. Pericardial tamponade, intracranial hemorrhage, and acute leg ischemia occurred in 10, 10, and 20% of patients, respectively. Also, 80% of patients developed acute kidney injury ≥ Kidney Disease: Improving Global Outcomes *(*KDIGO) 3 and 50% acute liver failure (i.e., SOFA liver ≥ 2) during their ICU stay. Seven (70%) patients required renal replacement therapy and seven (70%) patients survived ICU discharge. In-ICU deaths were attributed to brain death for two and multiple organ failure for one patient. Respective median durations of ECMO and mechanical ventilation support were 4 (1–6) and 5 (1–13) days. The median ICU length of stay was 12 (1–25) days. All infants survived (Fig. 1).

**Table 2:** ECMO management, in ICU complications, and outcomes of patients with amniotic fluid embolism rescued by VA-ECMO

	*N* = 10
*Before ICU peri-delivery hemorrhage*	10 (100)
Red blood cell transfusion, units	13 (4–32)
Fresh frozen plasma transfusion, units	13 (4–37)
Platelet transfusion, units	5 (0–30)
Fibrinogen administration, g	8 (0–18)
Hemostatic procedure	7 (70)
Surgical hysterectomy	5 (50)
Triple ligature	1 (10)
Selective arterial embolization	1 (10)
*ECMO*	
Pre-ECMO inotropic score, μg/kg/min	370 (55–1530)
Pre-ECMO lactate, mmol/L	12 (2–30)
Pre-ECMO left ventricular function, %	14 (0–40)
ECMO duration, days	4 (1–6)
Levosimendan during ECMO	1 (10)
ECMO-related complication	5 (50)
Pericardial tamponade	1 (10)
Intracranial hemorrhage	1 (10)
Acute leg ischemia	2 (20)
*In-ICU*	
Acute kidney injury ≥ KDIGO 3	8 (80)
Renal replacement therapy	7 (70)
Acute liver failure (i.e., SOFA liver ≥ 2)	5 (50)
Red blood cell transfusion, units	6 (0–19)
Fresh frozen plasma transfusion, units	9 (0–49)
Platelet transfusion, units	13 (0–75)
At least one ventilator associated pneumonia	3 (30)
*Outcomes*	
ECMO duration, days	4 (1–6)
In survivors, days	4 (3–6)
Inotrope duration, days	5 (1–8)
Mechanical ventilation duration, days	5 (1–13)
In survivors, days	6 (2–13)
ICU length of stay, days	12 (1–25)
In survivors, days	16 (5–25)
Maternal survival at ICU discharge	7 (70)
Infant survival	10 (100)

Data are expressed as n (%) or median (range)

*ECMO* extracorporeal membrane oxygenation, *ICU* intensive care unit, *KDIGO* Kidney Disease: Improving Global Outcomes, *SOFA* Sequential Organ Failure Assessment

**Fig. 2:**
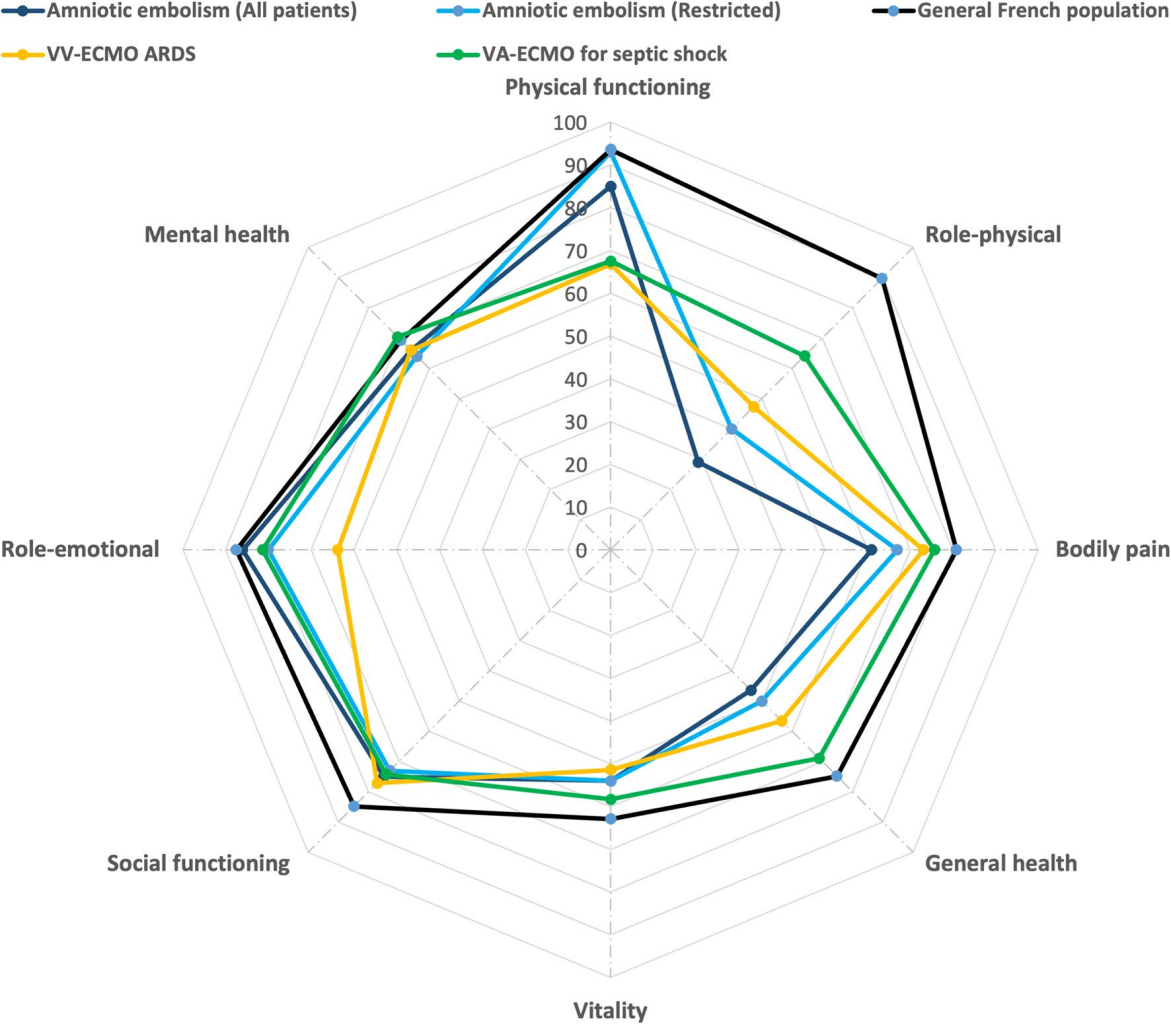
Comparison of mean SF-36 scores of AFE survivors treated by ECMO after a median follow-up of 40 months after intensive care unit discharge and their age- and sex-matched French control subjects [10], and 84 venovenous ECMO treated ARDS survivors [12], and 32 severe septic shock rescued by VA-ECMO [13]. Higher scores denote a better health-related quality of life. *ARDS* acute respiratory distress syndrome, *VV-ECMO* venovenous extracorporeal membrane oxygenation, *VA-ECMO* venoarterial extracorporeal membrane oxygenation

A long-term evaluation was conducted after a median of 44 (2–94) months after ICU discharge in seven patients. Among these 7 patients, 4 had returned to their previous work (60%), and 2 had a new pregnancy (but three patients had a hysterectomy during AFE episode). Severe motor sequellae were reported, related to femoral artery stenosis on the cannulation site for one patient and due to to acute leg ischemia leading to transfemoral amputation for a second one. All infants were in healthy conditions. SF-36 assessment HRQL is reported in Fig. 2. Compared with age- and sex-matched controls, our responding AFE survivors had significantly lower SF-36 physical domain scores (*p* < 0.01). Their psychological domain scores were comparable with those of the general population, except for their social functioning component, which was lower (*p* = 0.04). Their SF-36 physical aggregate scores were also significantly lower compared to age- and sex-matched controls (*p* < 0.01), while their mental aggregate scores were comparable. Compared to other populations rescued by either VV or VA ECMO, role- physical, bodily pain, and general health components were lower despite similar or better physical functioning components (Fig. 2). Two patients had an IES score ≥ of 30 (i.e, patients at risk for PTSD) 2 and 64 months after ICU discharge, respectively.

## Discussion

To our knowledge, this is the largest and most detailed follow-up study of catastrophic AFE patients requiring ECMO. AFE is a rare ECMO indication but is associated with a 70% survival rate at hospital discharge despite an extreme pre-ECMO severity. In this rare per-delivery complication, our results support the use of VA-ECMO despite intense DIC and active bleeding. Besides, physicians should be aware that these patients would need massive blood product transfusion during the first ECMO days. However, long-term HRQL was lower than age-matched controls and still profoundly impaired in the role-physical, bodily pain, and general health components when compared to other survivors rescued by ECMO.

The diagnosis of AFE is challenging as there are widely varying criteria for the diagnosis of AFE [5], most of AFE definitions are nonspecific, biological markers may not be specific to AFE [18], and exhaustive searches for alternative diagnoses are not uniformly done in a context of critically ill patients. For these reasons, we decided to restrict our inclusion criteria to the most recent definition proposed by Clark et al. [5]. These uniform diagnostic criteria help to prevent the inclusion of patients with alternative diagnoses such as hypovolemic shock secondary to postpartum hemorrhage, anesthetic accident, pulmonary thromboembolism, septic, and anaphylactic shock. For instance, we excluded 3 patients for whom AFE diagnosis was retained in the medical record without strictly fulfilling all of our AFE criteria. Two of them did not have DIC and one had fever, which could have been compatible with anaphylactic shock and septic shock, respectively. Several studies suggest a role for IGFBP-1 in the development of multiple organ dysfunction [19]. As detection of squamous cells into the maternal circulation or in the lungs requires procedural skill and may not be specific to AFE [20, 21], a high concentration of serum IGFBP-1 levels has been proposed for the diagnosis of AFE [18]. However, serum markers could be sometimes difficult to interpret since massive transfusion often occurred, distorting maternal serum dosages. Because of the lack of a systematic measure of IGFBP-1 and inconsistent quantitative measurement in all pregnant women on ECMO during the study period, this biomarker was not retained as an inclusion criterion.

Extracorporeal life support has been successfully used in pregnant and postpartum patients for a variety of indications [22]. Despite an extreme severity at the time of ECMO initiation, the 70% survival rate observed in this series is similar to the case-fatality reported in other case series (11–43%) with patients with lower severity [1, 18]. By aggressively treating bleeding events and judiciously managing systemic anticoagulation, ECMO appears adequate to rescue very severe AFE. Indeed, AFE is a self-resolving disease affecting the pulmonary and cardiovascular systems with most death and complications occurring during the first 24 h [1]. Our results reinforce that high bleeding risk should not be seen as an absolute contraindication to use ECMO [23]. However, ECMO management should be wisely adapted to these complex situations with DIC, thrombocytopenia, and frequent ongoing bleeding. An anticoagulation-free ECMO strategy, which combines no heparin bolus at cannulation and no anticoagulation as long as there are bleeding and/or DIC appears mandatory. This strategy on VA-ECMO is feasible and has been reported with other ECMO indications such as trauma [24] or refractory pulmonary embolism despite thrombolysis [25]. As reported in our results, massive transfusion of fresh frozen plasma, platelets, red blood cell packs, and fibrinogen, as well as arterial embolization or surgical hemostasis is frequently needed before or during cardiopulmonary support by VA-ECMO.

The favorable survival of these very severe patients should not overshadow that the long-term HRQL was still impaired compared with that of sex-and age-matched controls, especially concerning SF-36 general health, vitality, role-physical and social functioning, while mental health and role-emotional were considered satisfactory. Although differences in case mixes make comparisons between series difficult we observed that our patient’s SF-36 scores were worse than those reported by ARDS or septic shock survivors rescued by VV or VA-ECMO, respectively [12, 13]. Muscle weakness, chronic fatigue, and pain still significantly impact work or daily activities after a median of 44 months after ICU discharge. Previous studies also highlighted that women who give birth with severe obstetrical adverse events are at higher risk of long-term physical and mental health sequels compared to uncomplicated obstetrical populations [26]. In our population, only 60% of these young women returned to their previous work. These results highlight the urgent need for studies focusing on the establishment of customized, patient-centered, rehabilitation programs that could help improve long-term postpartum health-related quality of life [27–29].

We are aware that our study has limitations. First, AFE is a relatively rare disease that has limited our sample size. Second, our patients were treated in high-volume, experienced ECMO centers. Because better post-ECMO outcomes have been reported in such centers [30], caution is required when extrapolating these results to less-experienced ECMO centers. Third, the sole strict clinical-biological definition of AFE may have led to underreporting some cases. Therefore, we cannot rule out that more AFE cases could have been included with other AFE definitions or based on serum biomarkers. Lastly, the self-assessed, persistently impaired physical health and vitality might not be specific to ECMO itself but may represent sequelae of any severe disease requiring high doses of vasopressors, transfusion, and prolonged ICU stays as well as psychological and somatic sequelae after a highly complicated delivery.

## Conclusion

In conclusion, the survival of ECMO-rescued pregnant women with catastrophic AFE in our experienced centers was 70%. Ongoing bleeding, need for massive transfusion and DIC should not refrain physicians to early contact a Mobile Circulatory Assistance Units to prompty initiate VA-ECMO in these severely ill patients with cardiopulmonary dysfunction and high bleeding risk. However, general health, vitality, and role-physical impairment reported after long-term follow-up emphasizes the importance to integrate these young patients into customized, patient-centered, rehabilitation programs after ICU discharge.

## Data Availability

Not applicable.

## Abbreviations

AFE: Amniotic fluid embolism
DIC: Disseminated intravascular coagulopathy
ARDS: Acute respiratory distress syndrome
ICU: Intensive care unit
IGFBP-1: Insulin-like growth factor binding protein-1
HRQOL: Health-related quality of life
PTSD: Post-traumatic stress disorder
SAPS II: Simplified acute physiology score II
SOFA: Sequential organ-failure assessment
VA-ECMO: Venoarterial extracorporeal membrane oxygenation
